# Racial and Ethnic Disparities in Structural Measures of Brain Aging: A Systematic Review

**DOI:** 10.1093/geroni/igaa057.2826

**Published:** 2020-12-16

**Authors:** Sara Godina, Indira Turney, Laura Zahodne, Danielle Beatty Moody, Kacie Deters, Jennifer Manly, Andrea Rosso

**Affiliations:** 1 University of Pittsburgh Graduate School of Public Health, Pittsburgh, Pennsylvania, United States; 2 Columbia University Medical Center, New York, New York, United States; 3 University of Michigan, Ann Arbor, Michigan, United States; 4 University of Maryland Baltimore County, Baltimore, Maryland, United States; 5 Stanford University, Palo Alto, California, United States; 6 Columbia University, New York, New York, United States; 7 School of Public Health, University of Pittsburgh, Pittsburgh, Pennsylvania, United States

## Abstract

Examining the neural correlates underlying racial differences in cognitive functioning is necessary to help clarify mechanisms and to improve the generalizability of findings related to brain-behavior relationships. This systematic review aimed to determine whether there are racial differences in structural markers of brain aging among community-dwelling, neurologically healthy adults aged 18 and older. We identified studies (n=5,399) from searches in Ovid Medline, Ovid PsychINFO, and Elsevier EMBASE published until February 13, 2020. We included original articles that examined structural markers of brain aging and neuropathology across individuals from more than one race or ethnicity. These measures included magnetic resonance imaging, positron emission tomography amyloid and tau, cerebrospinal fluid amyloid, tau, and neurofilament light chain and computed tomography. Two reviewers independently screened full-text articles. We will present ongoing results and discuss the current state of knowledge, quality of existing studies, and gaps in the literature and highlight potentially key next steps.

